# *In vivo* detection of γ-glutamyl-transferase up-regulation in glioma using hyperpolarized γ-glutamyl-[1-^13^C]glycine

**DOI:** 10.1038/s41598-020-63160-y

**Published:** 2020-04-10

**Authors:** Georgios Batsios, Chloé Najac, Peng Cao, Pavithra Viswanath, Elavarasan Subramani, Yutaro Saito, Anne Marie Gillespie, Hikari A. I. Yoshihara, Peder Larson, Shinsuke Sando, Sabrina M. Ronen

**Affiliations:** 1 Department of Radiology and Biomedical Imaging, Mission Bay Campus, University of California, 1700 4th Street, Byers Hall, 94158 San Francisco, CA United States; 2 Department of Chemistry and Biotechnology, The University of Tokyo, Tokyo, Japan; 3 Laboratory for Functional and Metabolic Imaging, EPFL, Lausanne, Switzerland

**Keywords:** Cancer imaging, Cancer metabolism, Tumour biomarkers, Cancer

## Abstract

Glutathione (GSH) is often upregulated in cancer, where it serves to mitigate oxidative stress. γ-glutamyl-transferase (GGT) is a key enzyme in GSH homeostasis, and compared to normal brain its expression is elevated in tumors, including in primary glioblastoma. GGT is therefore an attractive imaging target for detection of glioblastoma. The goal of our study was to assess the value of hyperpolarized (HP) γ-glutamyl-[1-^13^C]glycine for non-invasive imaging of glioblastoma. Nude rats bearing orthotopic U87 glioblastoma and healthy controls were investigated. Imaging was performed by injecting HP γ-glutamyl-[1-^13^C]glycine and acquiring dynamic ^13^C data on a preclinical 3T MR scanner. The signal-to-noise (SNR) ratios of γ-glutamyl-[1-^13^C]glycine and its product [1-^13^C]glycine were evaluated. Comparison of control and tumor-bearing rats showed no difference in γ-glutamyl-[1-^13^C]glycine SNR, pointing to similar delivery to tumor and normal brain. In contrast, [1-^13^C]glycine SNR was significantly higher in tumor-bearing rats compared to controls, and in tumor regions compared to normal-appearing brain. Importantly, higher [1-^13^C]glycine was associated with higher GGT expression and higher GSH levels in tumor tissue compared to normal brain. Collectively, this study demonstrates, to our knowledge for the first time, the feasibility of using HP γ-glutamyl-[1-^13^C]glycine to monitor GGT expression in the brain and thus to detect glioblastoma.

## Introduction

Redox homeostasis is essential for managing oxidative stress and ensuring cell survival. Cancer cells, in particular, are often characterized by high levels of oxidative stress. This oxidative stress is the result of reactive oxygen species (ROS) that accumulate due to a variety of factors, including rapid cell proliferation, hypoxia, metabolic reprogramming and oncogenic signaling. The tripeptide glutathione (L-γ-glutamyl-L-cysteinyl-glycine, GSH) is the most abundant, non-enzymatic antioxidant molecule present in mammalian cells and plays a crucial role in regulating tumor oxidative stress due to its role in reducing ROS^1,2^. Several tumor types, including primary glioblastomas (GBMs), are characterized by elevated GSH levels, especially under hypoxic conditions^3^. Elevated GSH levels and reduced oxidative stress have also been linked to resistance to chemotherapy in GBMs^4^.

GSH import is a critical step in the maintenance of both intracellular and extracellular redox status^5,6^. Although GSH can be transported out of cells and into the extracellular environment, most cells are incapable of importing intact GSH^7^. Instead, GSH is first degraded to its constituent amino acids^7,8^ and the released amino acids are then transported into the cell and used as substrates for *de novo* GSH synthesis^6^. Specifically, GSH degradation is initiated by cleavage of the gamma-glutamyl bond^9^ via the cell surface-bound glycoprotein gamma-glutamyl transpeptidase (GGT) whose catalytic site faces the extracellular environment^7,10–12^. After removal of the glutamyl group from GSH, cysteinylglycine is cleaved by a membrane-bound dipeptidase to the constituent amino acids cysteine and glycine. In addition to facilitating GSH import, GGT activity, therefore, increases the intracellular availability of cysteine, which is often rate-limiting for GSH synthesis^13,14^. Due to its role in GSH homoeostasis, GGT overexpression and elevated GSH levels often go hand-in-hand.

It has been demonstrated that GGT is overexpressed in many human malignancies including primary GBMs, whereas GGT expression is low in normal brain astrocytes and neurons^4,15–19^. The high level of expression of GGT in glioblastoma and its presence on the outer cell membrane make it an attractive molecular target for assessing redox and imaging GBMs. Non-invasive methods of monitoring GGT using radiolabeled or fluorescence probes have been previously described^18,20–22^. However radiolabeled agents do not provide information regarding enzyme activity as they accumulate at the tumor site by diffusion, and the utility of fluorescent probes is limited by the depth of fluorescent light penetration through the skull. An alternative approach is the use of ^13^C magnetic resonance spectroscopy/imaging (MRS/I) combined with a hyperpolarized (HP) molecular probe. Dissolution dynamic nuclear polarization (dDNP) is a versatile method that allows hyperpolarization of nuclear spins, leading to >10,000-fold enhancement in the signal to noise ratio (SNR) of polarized ^13^C-labeled probes compared to the non-polarized agent. This allows for rapid, non-invasive, pathway-specific, real-time monitoring of metabolic and physiological processes^23^. Over the past decade many HP probes have been developed and used successfully *in vivo* to image normal and diseased tissues^24^, including *in vivo* in patients^25–28^.

Previous studies have shown^16,29,30^ that GGT can cleave other peptides carrying a gamma-glutamyl motif, besides GSH. Based on this logic, Nishihara *et al*.^31^ developed HP γ-glutamyl-[1-^13^C]glycine, which upon cleavage by GGT releases [1-^13^C]glycine, as a real-time, non-invasive probe of GGT activity *in vivo*. HP γ-glutamyl-[1-^13^C]glycine has been used to evaluate GGT activity in kidneys and in a subcutaneous ovarian carcinoma xenograft model^31–33^. However, to the best of our knowledge, the utility of this HP probe for monitoring glioblastoma tumor burden in the brain *in vivo* has not yet been investigated.

The goal of our study was, therefore, to evaluate the feasibility of using HP γ-glutamyl-[1-^13^C]glycine to monitor GGT activity *in vivo* in healthy rat brain and in an orthotopic glioblastoma rat model. We found that the ratio of HP [1-^13^C]glycine to HP γ-glutamyl-[1-^13^C]glycine was significantly higher in tumor-bearing rats when compared to healthy tumor-free control animals, and that in tumor-bearing rats glycine production was higher in the tumor region compared to normal-appearing surrounding brain. Importantly, our imaging findings were consistent with higher GGT expression and higher GSH levels in glioblastoma tissues compared to normal brain. Taken together, our study has identified HP γ-glutamyl-[1-^13^C]glycine as a potential non-invasive probe of GGT activity in orthotopic glioblastoma *in vivo*.

## Results

### Characterization of HP γ-glutamyl-[1-^13^C]glycine

**Figure 1:**
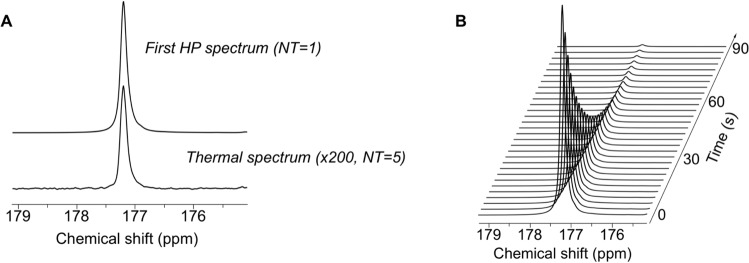
Characterization of the hyperpolarized (HP) probe γ-glutamyl-[1-^13^C]glycine **(A)** γ-glutamyl-[1-^13^C]glycine HP spectrum (top) and thermal equilibrium spectrum (bottom) at 11.7T illustrating that the DNP method leads to a polarization level (back calculated to time of dissolution) of 22.9 ± 6.7%. **(B)** Typical stack plot of ^13^C MR spectra of HP γ-glutamyl-[1-^13^C]glycine in solution acquired at 11.7T and used to calculate the T_1_ (temporal resolution 3 s).

HP γ-glutamyl-[1-^13^C]glycine was synthesized as previously described^31^, dissolved in NaOH solution and mixed with OX063 and glycerol. Following polarization for 1.5 h, the resonance of γ-glutamyl-[1-^13^C]glycine was detected at 177.5 ppm with a polarization level (back calculated to time of dissolution) of 22.9 ± 6.7% (n = 3) when compared to the thermal spectrum (Fig. 1A). T_1_ values of HP γ-glutamyl-[1-^13^C]glycine were measured in solution at 3T and 11.7T by fitting the dynamically acquired HP ^13^C spectra (Fig. 1B) with a monoexponential curve after correcting for flip angle. The T_1_ of HP γ-glutamyl-[1-^13^C]glycine was 33 ± 3.5 s at 3T (n = 3) and 18 ± 2.5 s (n = 3) at 11.7T, consistent with an expected reduction in the T_1_ of ^13^C carbonyl groups at higher field and were comparable with published values^31^.

### *In vivo* HP studies in healthy and glioblastoma-bearing rats at 3T

**Figure 2:**
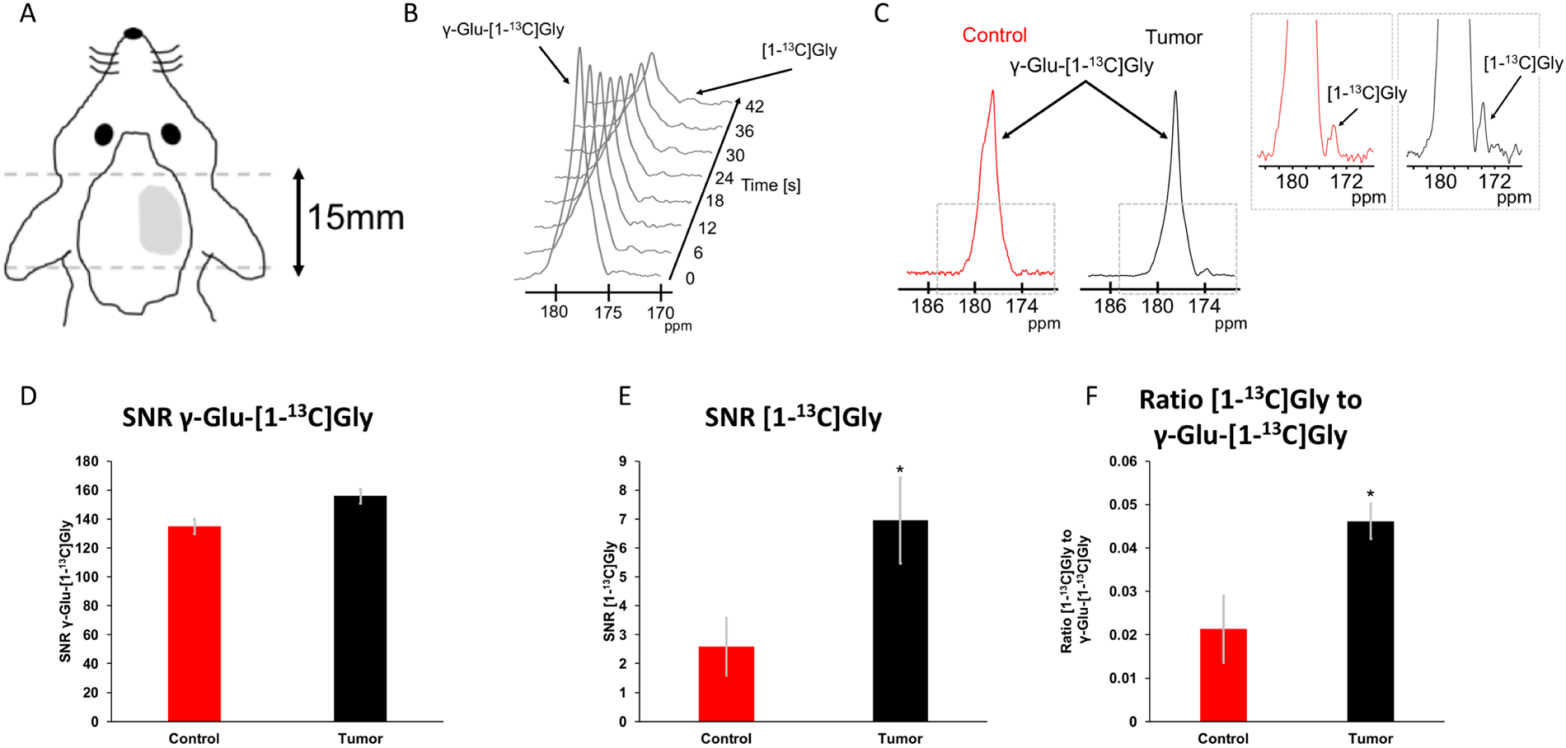
Representative HP ^13^C data acquired on 3T. **(A)** Illustration of HP data acquisition using a 15 mm slab. **(B)** Stack plot of hyperpolarized ^13^C data acquired from a tumor-bearing rat, showing decay of HP γ-glutamyl-[1-^13^C]glycine (γ-Glu-[1-^13^C]Gly) and production of HP [1-^13^C]glycine ([1-^13^C]Gly) as a function of time (temporal resolution 6 sec). **(C)** Sum spectra from the dynamic acquisition and zoom of spectra showing HP [1-^13^C]Gly production. **(D)** Quantification of γ-Glu-[1-^13^C]Gly SNR (134.8 ± 16.5 a.u. vs 155.7 ± 31.8 a.u. for control and tumor animals respectively). **(E)** Quantification of [1-^13^C]Gly SNR (2.58 ± 1.00 a.u. vs 6.96 ± 1.49 a.u. for control and tumor animals respectively). **(F)** [1-^13^C]Gly-to-γ-Glu-[1-^13^C]Gly ratios (0.021 ± 0.008 a.u. vs 0.046 ± 0.004 a.u. for control and tumor animals respectively). Red: Healthy control; Black: tumor-bearing animal. Animals per group = 7. *p < 0.05.

Dynamic HP ^13^C slab acquisitions using a spectral spatial slice selective scheme were performed on glioblastoma-bearing rats once the tumor reached a volume of 0.27 ± 0.06 cm3 (Fig. 2A,B). Tumor-free healthy rats were used as controls. The signal from the substrate HP γ-glutamyl-[1-^13^C]glycine could be detected in both healthy and tumor-bearing animals (Fig. 2C). Comparison of HP γ-glutamyl-[1-^13^C]glycine SNR showed no statistically significant difference between tumor-bearing and healthy rats (134.8 ± 16.5 a.u. vs 155.7 ± 31.8 a.u. for control and tumor animals respectively; p = 0.6; n = 7; Fig. 2D), indicating no significant difference in substrate delivery. The SNR of the product [1-^13^C]glycine (173.7 ppm) was below detection in individual spectra of the slab dynamic acquisition (Fig. 2B), but the sum of the dynamic ^13^C spectra showed improved SNR and [1-^13^C]glycine could be readily detected in all tumor-bearing animals and in some tumor-free healthy animals (Fig. 2C). Importantly however, the SNR of HP [1-^13^C]glycine in tumor-bearing rats was significantly higher relative to healthy rats (2.58 ± 1.00 a.u. vs 6.96 ± 1.49 a.u. for control and tumor animals respectively; p = 0.046; n = 7; Fig. 2E) as was the ratio of HP [1-^13^C]glycine to γ-glutamyl-[1-^13^C]glycine (0.021 ± 0.008 a.u. vs 0.046 ± 0.004 a.u. for control and tumor animals respectively; p = 0.027; n = 7; Fig. 2F). Additionally, the ratio of [1-^13^C]glycine to γ-glutamyl-[1-^13^C]glycine was positively correlated with the tumor fraction in the slab (R² = 0.8188; Supplementary Fig. 1).

**Figure 3:**
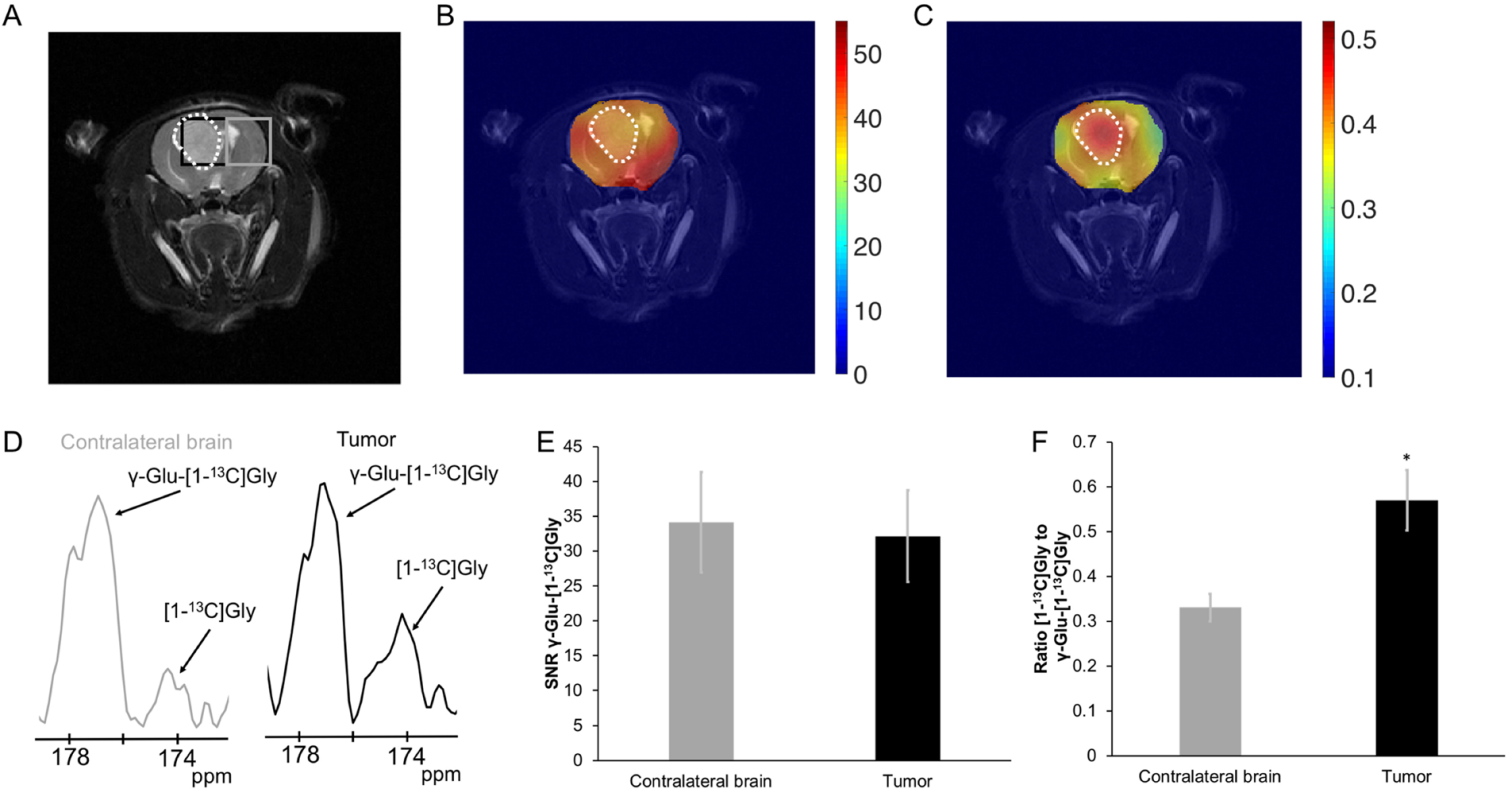
Representative HP ^13^C imaging acquired at 3T. **(A)** Representative T_2_-weighted anatomical image from a tumor-bearing animal. **(B)** Heat map of γ-glutamyl-[1-^13^C]glycine (γ-Glu-[1-^13^C]Gly) SNR illustrating homogeneous distribution of substrate in the brain. **(C)** Heat maps of the ratio of maximum [1-^13^C]glycine ([1-^13^C]Gly) to maximum γ-Glu-[1-^13^C]Gly showing that metabolism in the tumor region is higher than in normal brain. White dotted line outlines the tumor. **(D)** Representative spectra collected from contralateral brain (gray) and tumor (black) voxels. **(E)** Quantification of γ-Glu-[1-^13^C]Gly SNR. γ-Glu-[1-^13^C]Gly SNR in normal brain and tumor values are 34.1 ± 7.2 a.u. and 32.1 ± 6.6 a.u. respectively. **(F)** [1-^13^C]Gly-to-γ-Glu-[1-^13^C]Gly ratios: 0.33 ± 0.03 a.u. vs 0.57 ± 0.07 a.u. for normal-appearing brain and tumor respectively. Gray: Contralateral brain; Black: Tumor. Animals per group = 4. *p < 0.05.

Next, we examined the spatial distribution of HP γ-glutamyl-[1-^13^C]glycine in a small sample of U87 glioblastoma-bearing rats (Fig. 3A) using a spectral -spatial 2D echo-planar spectroscopic imaging (EPSI) acquisition scheme. Metabolic heat maps (Fig. 3B,C) illustrate that the substrate HP γ-glutamyl-[1-^13^C]glycine was evenly distributed throughout the brain (Fig. 3B) while the ratio [1-^13^C]glycine to γ-glutamyl-[1-^13^C]glycine was higher in the tumor (Fig. 3C). Similar to the data collected from the slab acquisitions, spectra collected from the tumor voxel presented a higher amount of [1-^13^C]glycine compared to contralateral normal-appearing brain voxel (Fig. 3D). Moreover, the SNR of HP γ-glutamyl-[1-^13^C]glycine was not significantly different in regions of interest (ROI) comprising tumor tissue or normal-appearing brain (34.1 ± 7.2 a.u. vs 32.1 ± 6.6 a.u. for normal-appearing brain and tumor respectively; p = 0.86; n = 4; Fig. 3E). The ratio of HP [1-^13^C]glycine to γ-glutamyl-[1-^13^C]glycine evaluated in the same ROIs was higher in tumor compared to normal-appearing brain (0.33 ± 0.03 a.u. vs 0.57 ± 0.07 a.u. for normal-appearing brain and tumor respectively; p = 0.046; n = 4, Fig. 3F).

### *Ex vivo* evaluation of GGT enzyme expression and GSH levels in glioblastoma and normal brain

**Figure 4:**
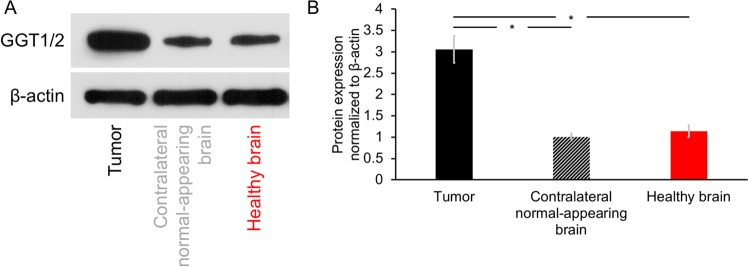
GGT enzyme expression evaluated by western blot assay. **(A)** Cropped western blots showing higher expression of γ-glutamyl-transferase 1 and 2 (GGT1/2) in glioma tumor compared to contralateral normal-appearing brain tissue and healthy brain tissue. β-actin was used as loading control. Complete blots can be seen in Supplementary Fig. 2. **(B)** Quantification of GGT levels for the three groups. Protein expression normalized to β-actin values: 3.06 ± 0.31 a.u., 1.00 ± 0.08 a.u. and 1.14 ± 0.14 a.u. for tumor, contralateral normal-appearing brain and healthy brain respectively. Black: tumor; Striped black bar: Contralateral normal-appearing brain; Red: Healthy brain. *p < 0.05.

GGT expression was previously shown to be higher in glioblastoma relative to normal brain^17^. In order to confirm that the higher production of HP [1-^13^C]glycine from HP γ-glutamyl-[1-^13^C]glycine in tumor relative to normal brain was linked to higher GGT expression, we examined GGT levels by western blotting in tumor and contralateral normal-appearing brain tissue from tumor-bearing rats. Brain tissue isolated from tumor-free healthy controls was also examined. As shown in Fig. 4A, glioblastoma tumor tissues showed higher expression of the GGT isoforms GGT1 and GGT2 compared to contralateral normal-appearing or tumor-free healthy brain tissues (3.06 ± 0.31 a.u., 1.00 ± 0.08 a.u. and 1.14 ± 0.14 a.u. for tumor, contralateral normal-appearing brain and healthy brain respectively; p = 0.03 tumor versus contralateral normal-appearing brain and p = 0.02 tumor versus healthy brain; Fig. 4A,B, Supplementary Fig. 2).

**Figure 5:**
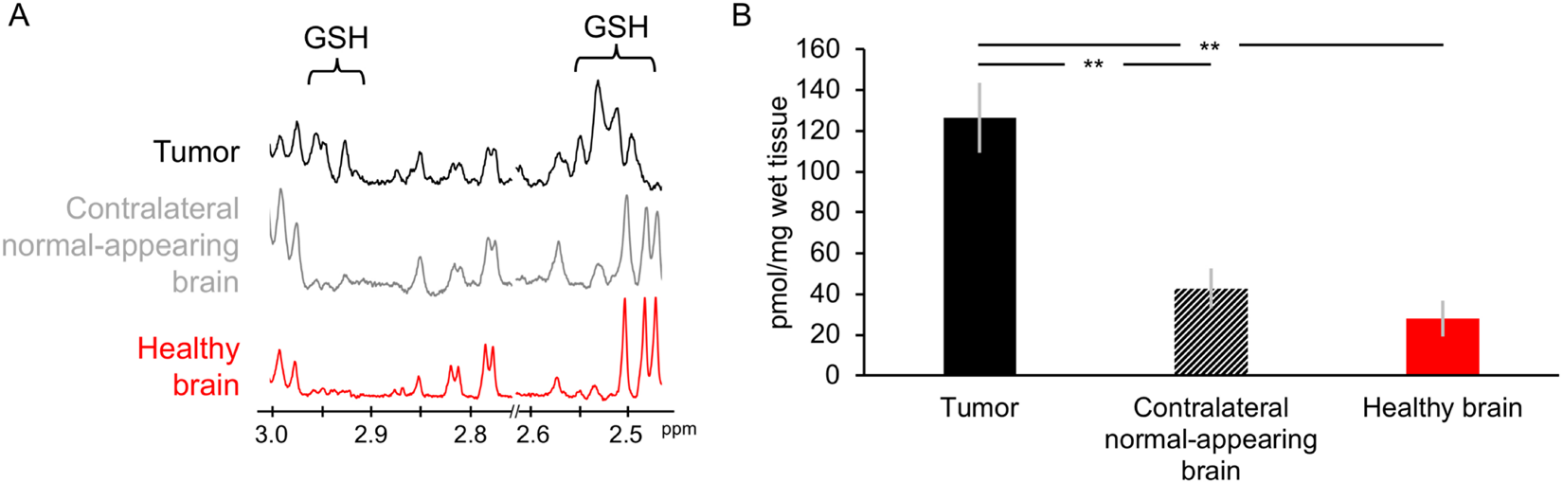
GSH levels in tumor, contralateral normal-appearing brain tissue and healthy brain tissues evaluated by MRS of extracts. **(A)** Typical 500 MHz ^1^H MRS spectrum of the aqueous fraction of tumor, contralateral normal-appearing brain and healthy brain where the GSH regions are highlighted. **(B)** Quantification of GSH levels. 126.6 ± 16.3pmol, 42.8 ± 9.1pmol and 28.0 ± 8.0pmol per mg of wet tissue of tumor, contralateral normal-appearing brain and healthy brain respectively. Black: tumor; Striped black bar: Contralateral normal-appearing brain; Red: Healthy brain. **p < 0.01.

In terms of function, GGT plays a key role in maintaining cellular GSH levels. To assess whether higher tumor GGT activity was linked to higher GSH levels, we measured GSH levels by ^1^H-MRS in extracts from U87 tumors, contralateral normal-appearing brain and healthy normal brain isolated from tumor-free animals. Representative ^1^H-MRS spectra are shown in Fig. 5A. Quantification of the data indicated that GSH levels (126.6 ± 16.3pmol, 42.8 ± 9.1pmol and 28.0 ± 8.0pmol per mg of wet tissue of tumor, contralateral normal-appearing brain and healthy brain respectively) were significantly higher in U87 tumors compared to contralateral normal brain (p = 0.006; n = 5) and compared to healthy brain tissue (p = 0.004; n = 3, Fig. 5B).

## Discussion

HP γ-glutamyl-[1-^13^C]glycine has previously been used to assess GGT activity in kidneys and in an ovarian carcinoma model^31–33^. The goal of this study was to evaluate the feasibility of using HP γ-glutamyl-[1-^13^C]glycine to non-invasively probe GGT expression in glioblastoma in the rat brain *in viv*o. We have demonstrated, to our knowledge for the first time, that the dynamic, real-time conversion of HP γ-glutamyl-[1-^13^C]glycine to [1-^13^C]glycine can be monitored *in vivo* in normal rat brain and in tumor-bearing animals. Furthermore, in tumor-bearing animals we were able to show, using 2D EPSI imaging, that elevated [1-^13^C]glycine production was localized to the tumor region. Importantly, our metabolic imaging data are linked to elevated GGT expression and elevated GSH levels in tumor tissue relative to contralateral normal-appearing brain and healthy normal brain tissues. Collectively, these results identify HP γ-glutamyl-[1-^13^C]glycine as a novel, non-invasive probe that is associated with cellular redox in brain tumors.

Previous studies have evaluated the expression of GGT in normal human brain, biopsies from brain tumors of different grades including GBM, and GBM cell lines including U87^18,19^. GGT expression was over 4-fold higher in GBM when compared to normal brain. Furthermore, GGT expression in U87 cells was comparable to its expression in GBM patient biopsies^18^. These results point to the validity of our studies with the U87 model, and the significance of our findings for imaging GBM tumors in patients.

γ-glutamyl-[1-^13^C]glycine fits the essential technical requirements of a useful HP probe^24^. Thanks to localization of the GGT enzyme on the outer surface of the cell membrane, γ-glutamyl-[1-^13^C]glycine metabolism occurs in the extracellular space and not in the cytosol or mitochondria as is the case for most HP probes including HP [1-^13^C]pyruvate^24^ and [1-^13^C]dehydroascorbic acid (DHA)^34,35^, or [1,3-^13^C]acetoacetate^36–38^, respectively. This is a significant advantage as additional transport though the cell membrane would increase the time between HP injection and enzymatic reaction and therefore decrease SNR. Compared to other probes^24,39^, the liquid state polarization level (~23%) of γ-glutamyl-[1-^13^C]glycine was relatively high, and the T_1_ relaxation time at clinical field strength (3T) was reasonably long (~33 s), such that it was possible to probe the metabolism of γ-glutamyl-[1-^13^C]glycine *in vivo* in an orthotopic glioma model. The chemical shift separation of 4 ppm between substrate (γ-glutamyl-[1-^13^C]glycine; 177.7 ppm) and product ([1-^13^C]glycine; 173.7 ppm) was also sufficient to allow visualization of product formation at the spectral resolution of our *in vivo* studies at 3T. Finally, when considering suitability for clinical translation, our study observed no noticeable changes in breathing during and after infusion of HP γ-glutamyl-[1-^13^C]glycine (48.6 mM). This observation was also consistent with a previous and more detailed study conducted by some of the authors of this manuscript^31^: no physiological changes associated with γ-glutamyl-[1-^13^C]glycine infusion were observed when monitoring breathing, blood pressure and heart rate using a pneumatic pillow, an arterial catheter and an intra-arterial blood pressure sensor.

GGT is a membrane-bound enzyme facing the extracellular compartment. It can be detected in normal brain, but is significantly higher in tumor^19^. There was no significant difference in the SNR values of HP γ-glutamyl-[1-^13^C]glycine in orthotopic U87 tumor-bearing rats and tumor-free healthy controls, consistent with a similar delivery to both tissues. Also the EPSI imaging results showed no difference in the uptake of HP γ-glutamyl-[1-^13^C]glycine between tumor and healthy brain area. Low [1-^13^C]glycine production in the normal brain and a significantly higher production in the tumor region are therefore most likely a reflection of the elevated tumor GGT expression. However, we cannot exclude higher delivery of HP γ-glutamyl-[1-^13^C]glycine to the more permeable tumor region also contributing to higher glycine production. After GGT removes the glutamyl group from GSH, cysteinylglycine is cleaved by an extracellular dipeptidase to the constituent amino acids cysteine and glycine, and the released amino acids are transported into the cell via dedicated transporters. We did not observe any consistent asymmetry in the lineshape of glycine peak, however, considering our line width (20-30 Hz) and the relatively long T_1_ of glycine (45 s at 9.4T^31^) we cannot rule out that some of the HP γ-glutamyl-[1-^13^C]glycine is intracellular. Importantly however, the precise compartmental localization of glycine would not be expected to affect our conclusions regarding its association with tumor.

Our slab results indicating that HP [1-^13^C]glycine production is significantly higher in tumor-bearing animals point to the utility of HP γ-glutamyl-[1-^13^C]glycine for non-invasively monitoring GGT activity in orthotopic glioblastoma *in vivo*. However, the SNR of our dynamic slab spectra was limited. Further improvements should therefore be considered to improve the slab data acquisition scheme. For example, one approach could be to combine a variable flip angle pulse sequence with the multiband method used here. As previously described, this approach would utilize progressively increasing flip angles between excitations and for each metabolite. The flip angles would be optimized to account for T_1_ relaxation, prior RF excitations and metabolic conversion in order to improve the SNR of the metabolites^40^. Additionally, further improvements could be obtained through changes in hardware. The use of a ^13^C circular polarized coil can provide a square root of 2 SNR improvement over the linear polarized ^13^C volume coil used in this study^41,42^. In the case of the EPSI studies, the voxel size used (346.7 μl) is larger than reports in literature for *in vivo* preclinical studies utilizing, for example, HP pyruvate^43–48^. Further improvements of the SNR and therefore the spatial resolution of the 2D EPSI acquisition scheme could be achieved through the above-mentioned variable flip angle scheme or the use of a circular polarized volume coil. An alternative approach could be to apply a high flip angle pulse on [1-^13^C]glycine and utilize all its magnetization at the time point when the [1-^13^C]glycine signal is maximal based on the dynamic slab acquisition. This approach has been previously reported for other probes by us and others^44,49–51^.

GGT is a membrane-bound enzyme that plays a key role in the metabolism of GSH. GGT catalyzes the degradation of extracellular GSH, thereby allowing recovery of constituent amino acids, including cysteine, which is often rate-limiting, for subsequent intracellular GSH re-synthesis. Due to the role of GSH as the principal water-soluble antioxidant within the cell, GGT has traditionally been regarded as crucial to cellular protection against oxidative stress. Cancer cells, in particular, suffer from higher levels of oxidative stress relative to normal cells. GGT expression is accordingly higher in cancer, including in high-grade primary glioblastoma, and non-invasive analysis of GGT activity, therefore, is associated with tumor antioxidant levels. Here we quantified GSH *ex vivo*, however prior work has used optimized ^1^H MRS sequences to separate GSH from the overlapping glutamate and glutamine peaks and assess steady-state GSH levels in patients *in vivo*^52–54^. Consistent with our preclinical findings, these studies showed that in brain tumors GSH levels were higher than in normal-appearing brain^55,56^. In complementary HP studies probing metabolic fluxes in animal models, previous investigations have used HP DHA^34,35^ and HP [1,3-^13^C]acetoacetate^36–38^, to non-invasively assess cellular redox status. However, the clinical translation of HP DHA is limited by potential toxicity issues^35^ while the conversion of HP acetoacetate to HP β-hydroxybutyrate is a measure of mitochondrial redox status. Our study identifies HP γ-glutamyl-[1-^13^C]glycine as a promising probe that can report on GGT and associated cellular redox to provide complementary information to GSH.

Comparison of orthotopic U87 tumor-bearing rats to tumor-free healthy controls showed a significantly higher level of HP [1-^13^C]glycine in tumor-bearing rats that positively correlated with tumor fraction. Spatial localization demonstrated that HP [1-^13^C]glycine was elevated in tumor tissue compared to normal brain. Importantly, the lower level of HP [1-^13^C]glycine production in healthy rats precludes the possibility that the HP [1-^13^C]glycine detected in tumor animals was exclusively produced in the kidney^16^ and then delivered to the brain. This is an important consideration given that, in many cancers, GGT can cleave GSH in interstitial fluid and blood^4,57^. Considering previous work showing that the ratio of [1-^13^C]glycine to total HP signal (γ-glutamyl-[1-^13^C]glycine plus [1-^13^C]glycine) is positively correlated with GGT activity^31^, previous studies showing higher levels of GGT in U87 glioma cells compare to normal brain^19^, and our observation that higher HP [1-^13^C]glycine production in tumor was associated with higher GGT expression in tumor tissue, our results collectively point to the potential of γ-glutamyl-[1-^13^C]glycine as a HP probe to detect brain tumor *in vivo* in real time. Furthermore, tumor therapy, and in particular radiation, can lead to “pseudoprogression” – MRI changes that cannot easily distinguish between tumor recurrence and treatment effects^58–60^. Because HP γ-glutamyl-[1-^13^C]glycine detects a molecular event specific to tumor, it could also help in evaluating treatment outcome. Indeed, the potential of a GGT-specific probe for distinguishing tumor recurrence from post-radiation effects has already been demonstrated in an *ex vivo* study using a GGT specific fluorescent probe^18^.

In summary, our study identifies HP γ-glutamyl-[1-^13^C]glycine as a probe for monitoring GGT activity in orthotopic glioblastoma *in vivo*. Higher [1-^13^C]glycine production in the tumor relative to normal brain *in vivo* was associated with higher GGT expression and higher steady-state GSH in the tumor. Given the critical role of GGT in redox homeostasis, our findings add to the repertoire of methods that can help to non-invasively assess redox in the brain and in brain tumors, and to more clearly distinguish tumor from normal brain.

## Materials and Methods

### γ-glutamyl-[1-^13^C]glycine probe preparation and hyperpolarization

γ-glutamyl-[1-^13^C]glycine was synthesized as previously described^31^. For hyperpolarization, 2.9 M γ-glutamyl-[1-^13^C]glycine was dissolved in 6.7 M NaOH solution and mixed with 21 mM OX063 (Oxford Instruments, UK) and 9.5 μl glycerol. 67 μl aliquot of the above solution was polarized in HyperSense polarizer (3.35T, 1.4 K, Oxford Instruments, UK) for ~1.5 hours and when maximal polarization was achieved, the sample was rapidly dissolved in 4 ml of isotonic buffer (40 mM Tris-HCl (pH = 8), 3 μM ethylenediaminetetraacetic acid (EDTA), 22 μM HCl) at physiological temperature.

### Relaxation and polarization levels

Following dissolution, HP γ-glutamyl-[1-^13^C]glycine was rapidly transferred to a horizontal 3T (Bruker BioSpec 105 mm bore diameter, n = 3, TR = 3 s/FA = 10°) or a vertical 11.7T (INOVA, Agilent Technologies, n = 3, TR = 3 s/FA = 13°,) system to evaluate T_1_. Percent polarization was quantified at 11.7T (n = 3, TR = 300 s/FA = 90°/NR = 5). Spectra were processed and peaks quantified by integration using MestReNova (Mestrelab). For T_1_ determination peak integrals were corrected for flip angle and fit with a monoexponential curve. The polarization level in solution was evaluated by comparing the first hyperpolarized spectrum of the dynamic set to the corresponding thermal equilibrium spectrum after correction for flip angle and number of averages and then back calculating the value to the time of dissolution (20 to 25 s prior to first spectral acquisition).

### Animal studies

#### Orthotopic glioma model

U87 cells were received from the UCSF Brain Tumor SPORE Biorepository and were maintained in Dulbecco’s modified Eagle’s medium (DMEM) supplemented with 10% fetal calf serum, 2 mM glutamine, and 100 U/ml penicillin and streptomycin under normoxic conditions for no more than 30 passages before inoculation. The cell line was authenticated by short tandem repeat fingerprinting (Cell Line Genetics) within 6 months of the study. All animal studies were performed in accordance with the National Institutes of Health Guide for the Care and Use of Laboratory Animals and were approved by the University of California San Francisco Institutional Animal Care and Use Committee (IACUC Protocol No: AN170079). 17 male athymic nu/nu rats (5 weeks old) were investigated. 7 animals were used as age-matched tumor-free controls. In 10 animals (6 used for slab acquisition, 3 for imaging and 1 for both), tumors were generated by implanting U87 glioblastoma cells (3 × 10^5^) by intracranial injection as previously described^49^.

#### MRI and HP ^13^C MRS *in vivo* studies

All measurements were performed on a horizontal 3T scanner (BioSpec 105 mm bore diameter, Bruker) equipped with a dual-tuned ^1^H-^13^C volume coil (42 mm inner diameter, Bruker). Animals were anesthetized and maintained using isoflurane (1-2% in O_2_) and placed head first in the prone position. Animal breathing was monitored using a small animal breathing monitoring system (MR-compatible Small Animal Monitoring, SA Instruments, USA) during all acquisitions. Axial and sagittal anatomical T_2_-weighted images were recorded using a spin echo (TurboRARE) sequence (TE/TR = 64/3484 ms, FOV = 35 × 35 mm^2^, 256 × 256, slice thickness=1 mm, NA = 10) and used to evaluate tumor location and size. HP studies were performed following injection of 2.2 ml HP γ-glutamyl-[1-^13^C]glycine (prepared as described above) via a tail-vein catheter over 15 s. Dynamic ^13^C MR spectra were acquired from a 15 mm slab through the brain every 3 s using a flyback spectral-spatial pulse with 30° excitation on product ([1-^13^C]glycine) and 4° on substrate (γ-glutamyl-[1-^13^C]glycine) (Supplementary Fig. 3)^61^. In the case of ^13^C MRSI, a 2D flyback spectral-spatial echo-planar spectroscopic imaging (EPSI) pulse was used with the same frequency profile as for the slab to provide a spatial resolution of 5.375 × 5.375 × 12 mm^3^, a temporal resolution of 3 s, spectral resolution of 128 points over a spectral bandwidth of 25 ppm. In all cases the scans started 12 s after the start of the HP γ-glutamyl-[1-^13^C]glycine injection.

#### Data processing

Tumor size was measured as the sum of manually contoured tumor areas in each slice multiplied by slice thickness using in-house software^49^. The HP experiments were performed when the tumor volume reached a value of ~0.27 cm^3^. The HP ^13^C spectra were analyzed using MestreNova (Mestrelab, Spain). Each spectrum was individually apodized (line broadening = 5 Hz) and phased. Then all the spectra were summed. Resonances for product ([1-^13^C]glycine) and substrate (γ-glutamyl-[1-^13^C]glycine) were fit with a Lorentzian-Gaussian line shape and their integral was normalized to the standard deviation of the noise. In addition, ratios of [1-^13^C]glycine to γ-glutamyl-[1-^13^C]glycine were quantified for each animal. The imaging data were processed using in-house Matlab code. For each voxel at every time point, spectra were analyzed after a 3 Hz line broadening by determining the area under each peak by integration. Intensity heat maps were produced by interpolating the data using a Lanczos-2 kernel and normalized to noise, which was evaluated as the standard deviation of the real part of the signal in a voxel outside of the brain. These maps were used to generate the ratio of substrate to product metabolic map. The SNR of γ-glutamyl-[1-^13^C]glycine and the ratio of [1-^13^C]glycine to γ-glutamyl-[1-^13^C]glycine were evaluated in a 76.2 mm^3^ volume region of interest comprising of tumor or healthy brain tissue.

### Immunoblotting

Tumors and contralateral normal-appearing brain tissues from tumor-bearing animals and healthy brain tissue from tumor-free control animals were excised after the HP MR scan, snap-frozen in liquid nitrogen and stored at −80 °C until further investigation. γ-glutamyl-transferase 1 and 2 (GGT1/2) levels in all samples were quantified using western blotting with β-actin as a loading control as follows. Tissues were lysed using RIPA Buffer (ThermoFisher Scientific) supplemented with 1 μl/ml protease inhibitor cocktail set III (Calbiochem). Lysates normalized to wet tissue weight were then run on 4–20% gels (Bio-Rad) using the SDS-PAGE method and electrotransferred onto nitrocellulose membranes. Membranes were blocked with 5% milk in Tris-Buffered Saline Tween-20 (TBST) and incubated with the primary antibodies anti-GGT1/2 (Santa Cruz Biotechnology sc-393706) at 1:100 dilution and anti-β-actin (Cell Signaling #4970) at 1:5000 dilution overnight at 4°C. HRP-conjugated secondary antibodies (Cell Signaling #7074) at 1:3000 dilution were incubated for 60 min in TBST at room temperature. Immunocomplexes were visualized using ProSignal Pico (Genesee Scientific). Densitometry of the bands was performed using ImageJ software (NIH) to quantify protein expression.

### Analysis of tumor tissue

#### Tissue extraction

The levels of GSH were evaluated by ^1^H MRS using snap frozen tissue from tumor, normal-appearing brain tissue or healthy brain tissue (11 to 18 mg wet-weight). The tissues were homogenized in 400 μl ice cold phosphate buffer (PBS) with 1 μl/ml protease inhibitor cocktail set III (Calbiochem) in the presence of TissueLyser beads (TissueLyser LT, QIAGEN). Afterwards a dual-phase extraction method was followed^62^. Briefly, 10 ml ice cold methanol (Sigma-Aldrich) was added to the homogenized tissue. The solution was then vortexed and 10 ml of ice-cold chloroform (Acros Organics) added. After another vortexing, 10 ml of ice cold Milli-Q water was added and a final vortexing performed. Phase separation was achieved by centrifugation for 10 min at 3000 rpm at 4 °C, the phases were separated, and solvents removed by lyophylization. The aqueous phase was then reconstituted for MRS studies in 400 μl phosphate buffer (pH = 7.4) in deuterium oxide (Acros Organics).

#### MRS acquisition and data analysis

^1^H spectra of the aqueous phase of tissue extracts were recorded using a 500 MHz spectrometer (Bruker BioSpin) equipped with a triple resonance cryoprobe. The ^1^H spectra were acquired using a 90° flip angle, 3 s repetition time (TR) with 384 averages. In addition, fully relaxed ^1^H spectra were recorded and served to determine correction factors for saturation. The concentration of GSH was quantified by peak integration using MestReNova. The integrals were corrected for saturation, and normalized to mg wet tissue and to an external sodium 3-(trimethylsilyl)propionate-2,2,3,3-d4 (TSP; Sigma-Aldrich) reference of known concentration.

### Statistical analysis

All results are expressed as mean ± standard error of the mean (SEM). One-way ANOVA was used to assess the statistical significance of differences in HP ^13^C MRS data between tumor-bearing animals and controls. Paired two-tailed Student’s t-test with unequal variance was used to assess the statistical significance of differences in western blots and ^1^H MRS. A p-value less than 0.05 was considered as statistically significant.

## Supplementary information

Supplementary Information.

## Data Availability

Data generated during the current study are available from the corresponding author upon request.

## References

[1] Rappa G, Lorico A, Flavell RA, Sartorelli AC (1997). Evidence that the multidrug resistance protein (MRP) functions as a co-transporter of glutathione and natural product toxins. Cancer Res.

[2] Gomi A, Shinoda S, Masuzawa T, Ishikawa T, Kuo MT (1997). Transient induction of the MRP/GS-X pump and gamma-glutamylcysteine synthetase by 1-(4-amino-2-methyl-5-pyrimidinyl)methyl-3-(2-chloroethyl)-3- nitrosourea in human glioma cells. Cancer Res.

[3] Ogunrinu TA, Sontheimer H (2010). Hypoxia increases the dependence of glioma cells on glutathione. J Biol Chem.

[4] Hanigan MH, Frierson HF, Swanson PE, De Young BR (1999). Altered expression of gamma-glutamyl transpeptidase in human tumors. Human Pathology.

[5] Lewerenz J, Maher P (2011). Control of redox state and redox signaling by neural antioxidant systems. Antioxidants & redox signaling.

[6] Lu SC (2013). Glutathione synthesis. Biochimica et biophysica acta.

[7] Meister A, Anderson ME (1983). Glutathione. Annual review of biochemistry.

[8] Hanigan M (1998). H. gamma-Glutamyl transpeptidase, a glutathionase: its expression and function in carcinogenesis. Chemico-biological interactions.

[9] Tate SS, Meister A (1974). Interaction of gamma-glutamyl transpeptidase with amino acids, dipeptides, and derivatives and analogs of glutathione. The Journal of biological chemistry.

[10] Hanes CS, Hird FJ (1950). Synthesis of peptides in enzymic reactions involving glutathione. Nature.

[11] Griffith OW, Meister A (1979). Translocation of intracellular glutathione to membrane-bound gamma-glutamyl transpeptidase as a discrete step in the gamma-glutamyl cycle: glutathionuria after inhibition of transpeptidase. Proceedings of the National Academy of Sciences of the United States of America.

[12] Griffith OW, Bridges RJ, Meister A (1978). Evidence that the gamma-glutamyl cycle functions *in vivo* using intracellular glutathione: effects of amino acids and selective inhibition of enzymes. Proceedings of the National Academy of Sciences of the United States of America.

[13] DeBerardinis RJ, Chandel NS (2016). Fundamentals of cancer metabolism. Science Advances.

[14] Cairns RA, Harris IS, Mak TW (2011). Regulation of cancer cell metabolism. Nature reviews. Cancer.

[15] Fischer P, Scherberich JE, Schoeppe W (1990). Comparative biochemical and immunological studies on gamma-glutamyltransferases from human kidney and renal cell carcinoma applying monoclonal antibodies. Clinica chimica acta; international journal of clinical chemistry.

[16] Hanigan MH, Frierson HF (1996). Immunohistochemical detection of gamma-glutamyl transpeptidase in normal human tissue. The journal of histochemistry and cytochemistry: official journal of the Histochemistry. Society.

[17] Schafer C (2001). Gamma-glutamyl transferase expression in higher-grade astrocytic glioma. Acta oncologica (Stockholm, Sweden).

[18] Shi, B. *et al*. Enhanced γ-Glutamyltranspeptidase Imaging That Unravels the Glioma Recurrence in Post-radio/Chemotherapy Mixtures for Precise Pathology via Enzyme-Triggered Fluorescent Probe. *Frontiers in Neuroscience* **13**, 10.3389/fnins.2019.00557 (2019). 10.3389/fnins.2019.00557 PMC6554337 31213974

[19] Liu Y (2018). Visualizing glioma margins by real-time tracking of γ-glutamyltranspeptidase activity. Biomaterials.

[20] Urano Y (2011). Rapid cancer detection by topically spraying a gamma-glutamyltranspeptidase-activated fluorescent probe. Sci Transl Med.

[21] Khurana H (2015). Preclinical Evaluation of a Potential GSH Ester Based PET/SPECT Imaging Probe DT(GSHMe)- to Detect Gamma Glutamyl Transferase Over Expressing Tumors. PloS one.

[22] Miyata Y (2017). Intraoperative imaging of hepatic cancers using gamma-glutamyltranspeptidase-specific fluorophore enabling real-time identification and estimation of recurrence. Scientific reports.

[23] Ardenkjaer-Larsen JH (2003). Increase in signal-to-noise ratio of> 10,000 times in liquid-state NMR. Proceedings of the National Academy of Sciences of the United States of America.

[24] Chaumeil MM, Najac C, Ronen SM (2015). Studies of Metabolism Using (13)C MRS of Hyperpolarized Probes. Methods Enzymol.

[25] Park I (2018). Development of methods and feasibility of using hyperpolarized carbon-13 imaging data for evaluating brain metabolism in patient studies. Magn Reson Med.

[26] Miloushev VZ (2018). Metabolic Imaging of the Human Brain with Hyperpolarized (13)C Pyruvate Demonstrates (13)C Lactate Production in Brain Tumor Patients. Cancer Res.

[27] Nelson SJ (2013). Metabolic imaging of patients with prostate cancer using hyperpolarized [1-(1)(3)C]pyruvate. Sci Transl Med.

[28] Grist JT (2019). Quantifying normal human brain metabolism using hyperpolarized [1–13C]pyruvate and magnetic resonance imaging. NeuroImage.

[29] Hanes CS, Hird FJ, Isherwood FA (1952). Enzymic transpeptidation reactions involving gamma-glutamyl peptides and alpha-amino-acyl peptides. The Biochemical journal.

[30] Lieberman MW (1995). gamma-Glutamyl transpeptidase. What does the organization and expression of a multipromoter gene tell us about its functions? The American journal of pathology.

[31] Nishihara T (2016). Direct Monitoring of gamma-Glutamyl Transpeptidase Activity *In Vivo* Using a Hyperpolarized (13) C-Labeled Molecular Probe. Angewandte Chemie (International ed. in English).

[32] Frank, S. F., Yoshihara, H. A., Itoda, M., Sando, S. & Gruetter, R. In *Proceedings of ISMRM Annual Meeting (Paris)* 3066 (2018).

[33] Seki, T. *et al*. In *Proceedings of ISMRM Annual Meeting (Paris)* 3052 (2018).

[34] Keshari KR (2011). Hyperpolarized 13C dehydroascorbate as an endogenous redox sensor for *in vivo* metabolic imaging. Proceedings of the National Academy of Sciences of the United States of America.

[35] Timm KN (2017). Assessing Oxidative Stress in Tumors by Measuring the Rate of Hyperpolarized [1-13C]Dehydroascorbic Acid Reduction Using 13C Magnetic Resonance Spectroscopy. The Journal of biological chemistry.

[36] Miller JJ, Ball DR, Lau AZ, Tyler DJ (2018). Hyperpolarized ketone body metabolism in the rat heart. NMR Biomed.

[37] von Morze C (2018). Direct assessment of renal mitochondrial redox state using hyperpolarized (13) C-acetoacetate. Magn Reson Med.

[38] Najac C (2019). *In vivo* investigation of hyperpolarized [1,3-(13)C2]acetoacetate as a metabolic probe in normal brain and in glioma. Scientific reports.

[39] Hesketh RL, Brindle KM (2018). Magnetic resonance imaging of cancer metabolism with hyperpolarized (13)C-labeled cell metabolites. Current opinion in chemical biology.

[40] Xing, Y., Reed, G. D., Pauly, J. M., Kerr, A. B. & Larson, P. E. Optimal variable flip angle schemes for dynamic acquisition of exchanging hyperpolarized substrates. *Journal of magnetic resonance* (San Diego, Calif.: 1997) 234, 75–81, 10.1016/j.jmr.2013.06.003 (2013). 10.1016/j.jmr.2013.06.003 PMC3765634 23845910

[41] Chen CN, Hoult DI, Sank VJ (1983). Quadrature detection coils—A further √2 improvement in sensitivity. Journal of Magnetic Resonance (1969).

[42] Glover GH (1985). Comparison of linear and circular polarization for magnetic resonance imaging. Journal of Magnetic Resonance (1969).

[43] Peeters TH (2019). Imaging Hyperpolarized Pyruvate and Lactate after Blood-Brain Barrier Disruption with Focused. Ultrasound. ACS chemical neuroscience.

[44] Choi Y-S (2018). Hyperpolarized [1-13C] pyruvate MR spectroscopy detect altered glycolysis in the brain of a cognitively impaired mouse model fed high-fat diet. Molecular. Brain.

[45] Miller JJ (2018). 13C Pyruvate Transport Across the Blood-Brain Barrier in Preclinical Hyperpolarised MRI. Scientific reports.

[46] Chaumeil MM (2016). Hyperpolarized (13)C MR imaging detects no lactate production in mutant IDH1 gliomas: Implications for diagnosis and response monitoring. NeuroImage. Clinical.

[47] Park JM (2016). Volumetric spiral chemical shift imaging of hyperpolarized [2-13c]pyruvate in a rat c6 glioma model. Magnetic Resonance in Medicine.

[48] Mayer D (2011). Dynamic and high-resolution metabolic imaging of hyperpolarized [1-13C]-pyruvate in the rat brain using a high-performance gradient insert. Magnetic Resonance in Medicine.

[49] Radoul M (2016). MR Studies of Glioblastoma Models Treated with Dual PI3K/mTOR Inhibitor and Temozolomide:Metabolic Changes Are Associated with Enhanced Survival. Mol Cancer Ther.

[50] Park JM (2013). Metabolic response of glioma to dichloroacetate measured *in vivo* by hyperpolarized (13)C magnetic resonance spectroscopic imaging. Neuro-oncology.

[51] Suh EH (2019). *In vivo* assessment of increased oxidation of branched-chain amino acids in glioblastoma. Scientific reports.

[52] An L (2009). Measurement of glutathione in normal volunteers and stroke patients at 3T using J-difference spectroscopy with minimized subtraction errors. J Magn Reson Imaging.

[53] Wijtenburg SA (2019). Comparing the reproducibility of commonly used magnetic resonance spectroscopy techniques to quantify cerebral glutathione. Journal of Magnetic Resonance Imaging.

[54] Prinsen H, de Graaf RA, Mason GF, Pelletier D, Juchem C (2017). Reproducibility measurement of glutathione, GABA, and glutamate: Towards *in vivo* neurochemical profiling of multiple sclerosis with MR spectroscopy at 7T. Journal of Magnetic Resonance Imaging.

[55] Li Y (2015). Short-echo three-dimensional H-1 MR spectroscopic imaging of patients with glioma at 7 Tesla for characterization of differences in metabolite levels. Journal of magnetic resonance imaging: JMRI.

[56] Li Y, Park I, Nelson SJ (2015). Imaging tumor metabolism using *in vivo* magnetic resonance spectroscopy. Cancer J.

[57] Hanigan MH (2014). Gamma-glutamyl transpeptidase: redox regulation and drug resistance. Advances in cancer research.

[58] Zikou A (2018). Radiation Necrosis, Pseudoprogression, Pseudoresponse, and Tumor Recurrence: Imaging Challenges for the Evaluation of Treated Gliomas. Contrast media &. molecular imaging.

[59] Villanueva-Meyer JE, Mabray MC, Cha S (2017). Current Clinical Brain Tumor Imaging. Neurosurgery.

[60] Hygino da Cruz LC, Rodriguez I, Domingues RC, Gasparetto EL, Sorensen AG (2011). Pseudoprogression and pseudoresponse: imaging challenges in the assessment of posttreatment glioma. AJNR. American journal of neuroradiology.

[61] Larson PE (2008). Multiband excitation pulses for hyperpolarized 13C dynamic chemical-shift imaging. Journal of magnetic resonance (San Diego, Calif.: 1997).

[62] Izquierdo-Garcia JL (2015). IDH1 Mutation Induces Reprogramming of Pyruvate Metabolism. Cancer Res.

