# Supplementary material for: In vivo detection of γ-glutamyl-transferase up-regulation in glioma using hyperpolarized γ-glutamyl-[1-13C]glycine
Source: Sci Rep. 2020 Apr 10;10:6244. doi: 10.1038/s41598-020-63160-y (PMC7148357; doi:10.1038/s41598-020-63160-y)
Supplement: Supplementary file 1 — Supplementary Information. [file 41598_2020_63160_MOESM1_ESM.pdf]

In vivo detection of γ-glutamyl-transferase up-regulation in glioma using hyperpolarized γ-glutamyl-[1-^{13}C]glycine

Georgios Batsios[#], Chloé Najac[#], Peng Cao, Pavithra Viswanath, Elavarasan Subramani, Yutaro Saito, Anne Marie Gillespie, Hikari A.I. Yoshihara, Peder Larson, Shinsuke Sando, Sabrina M. Ronen*

Georgios Batsios[1#], Chloé Najac[1#], Peng Cao[1], Pavithra Viswanath[1], Elavarasan Subramani[1], Yutaro Saito[2], Anne Marie Gillespie[1], Hikari A.I. Yoshihara[3], Peder Larson[1], Shinsuke Sando[2], Sabrina M. Ronen✉[1]

[1]Department of Radiology and Biomedical Imaging, Mission Bay Campus, 1700 4th Street, Byers Hall, University of California, 94158, San Francisco, CA, United States

[2]Department of Chemistry and Biotechnology, The University of Tokyo, Tokyo, Japan

[3]Laboratory for Functional and Metabolic Imaging, EPFL, Lausanne, Switzerland

# Authors have contributed equally to this work.

* Correspondence and requests for materials should be addressed to S.M.R. (email: sabrina.ronen@ucsf.edu)

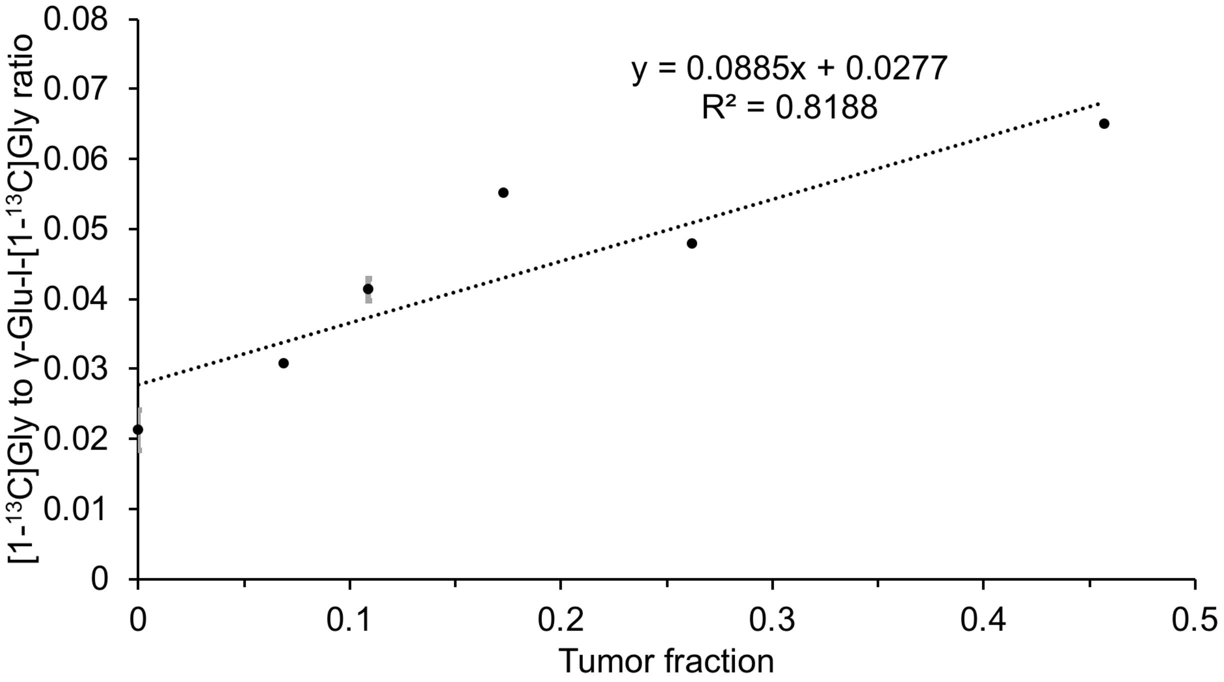

Figure S1. Correlation of [1-^{13}C]glycine to γ-glutamyl-[1-^{13}C]glycine ratio with tumor fraction in the slab.

A

GGT 1/2

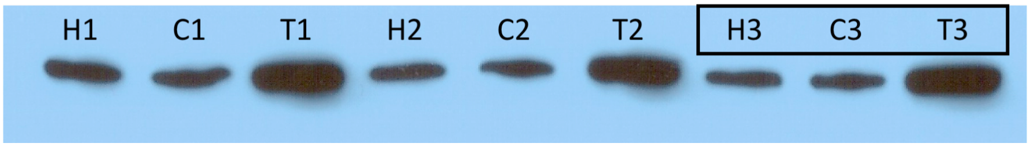

B

GGT 1/2

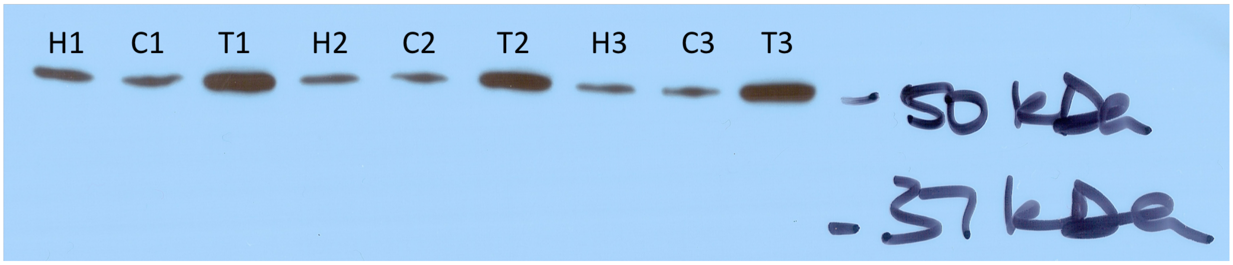

C

β-actin

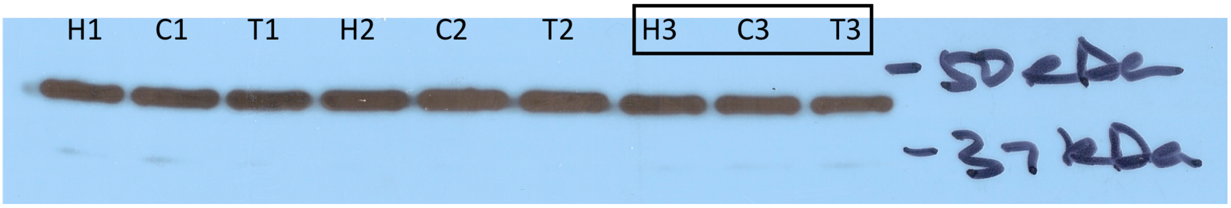

D

Sample	H1	C1	T1	H2	C2	T2	H3	C3	T3
**GGT1/2**	11195	9608	25115	8743	7935	22360	8505	7961	25573
**β-actin**	20489	22075	23125	25211	25810	24563	22801	21550	18374
**Ratio GGT1/2 to β-actin**	0.546	0.435	1.086	0.347	0.307	0.910	0.373	0.369	1.392

Figure S2. Uncropped western blots of GGT1/2 enzyme expression (A and B; different exposure times) and β-actin (C) for glioma tumor (samples marked as T1, T2, T3), contralateral normal-appearing brain tissue (samples marked as C1, C2, C3) and healthy brain tissue (samples marked as H1, H2, H3). The black box marks the samples that are presented in Figure 4. (D) Densitometric quantification of each band (from A and C) and intensity of each GGT1/2 band normalized to corresponding β-actin.

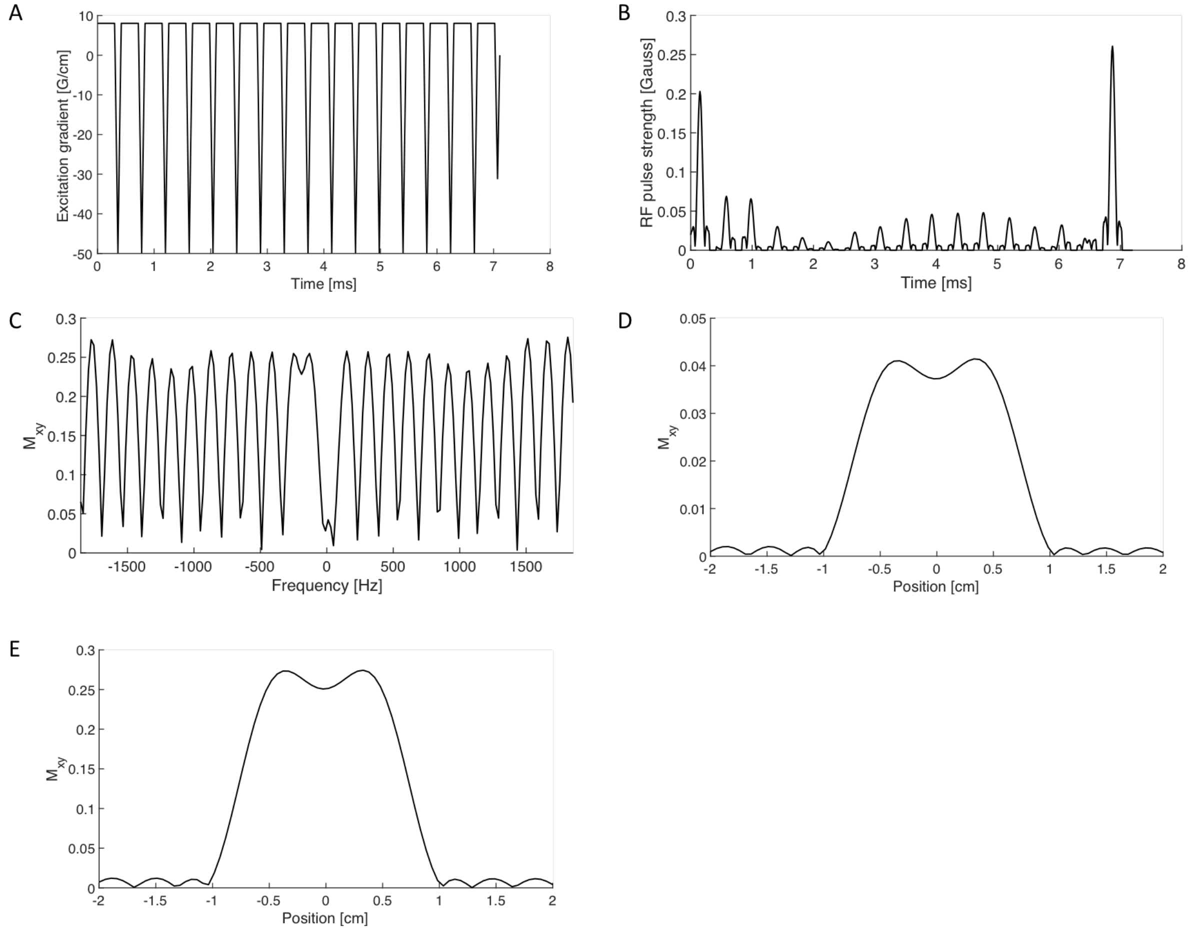

Figure S3. Spectral-spatial RF excitation pulse with independent flip angle control over two excitation bands. (A & B) RF and gradient waveforms. This 7.21ms pulse was designed for a 15mm slab, with the following resonances and their corresponding bandwidths and flip angles: 177.2ppm ± 1 ppm (γ-glutamyl-[1-^{13}C]glycine), 4°; 172.9 ppm ± 1 ppm ([1-^{13}C]glycine), 30°. (C) Spectral profile at the center of the slab (at 0 cm) and (D & E) spatial profile for γ-glutamyl-[1-^{13}C]glycine (D) and [1-^{13}C]glycine (E).
